# Vanillin Derivatives Reverse *Fusobacterium nucleatum*-Induced Proliferation and Migration of Colorectal Cancer Through E-Cadherin/β-Catenin Pathway

**DOI:** 10.3389/fphar.2022.841918

**Published:** 2022-03-04

**Authors:** Zhongkun Zhou, Yiqing Wang, Rui Ji, Dekui Zhang, Chi Ma, Wantong Ma, Yunhao Ma, Xinrong Jiang, Kangjia Du, Rentao Zhang, Peng Chen

**Affiliations:** ^1^ School of Pharmacy, Lanzhou University, Lanzhou, China; ^2^ The First Hospital of Lanzhou University, Lanzhou, China; ^3^ The Second Hospital of Lanzhou University, Lanzhou, China

**Keywords:** colorectal cancer, microbiome, vanillin derivatives, *Fusobacterium nucleatum*, 16S rRNA sequencing

## Abstract

Colorectal cancer (CRC) is a common clinical malignant tumor and closely related to intestinal microbiome disorders. Especially, *Fusobacterium nucleatum* (*F. nucleatum*) is one of the most prevalent pathogens in CRC. However, its change in CRC patients of Northwest China, an area with a high incidence of gastrointestinal tumors, is unclear, and therapeutic strategies targeting *F. nucleatum* remain unresolved. Here, fecal samples of healthy people and CRC patients were studied using 16S rRNA sequencing to explore microbial community alterations. Additionally, vanillin derivate (IPM711 and IPM712) intervention by coculture with CRC cells and potential mechanism were investigated. Results showed that intestinal microbial homeostasis was gradually dysregulated, and the abundance of *Fusobacterium* was higher in CRC patients. Moreover, IPM711 and IPM712 showed better anti-*F. nucleatum* activity than vanillin by increasing cell membrane permeability and destroying bacterial integrity. In addition, IPM711 and IPM712 could downregulate the expression of E-cadherin and β-catenin, thus, suppressing the migration of HCT116. Collectively, IPM711 and IPM712 have both anticolorectal cancer and anti-*F. nucleatum* activities, providing potential natural product drug candidates for microbe-targeted strategies for the treatment of CRC.

## Introduction

Colorectal cancer (CRC) is the third most prevalent cancer in both sexes combined, with an incidence of 6.1% and mortality of 9.2% (Bray et al., 2018). Development of CRC is a multifactorial process, among which gut microbes widely participated in the initiation, progression, metastasis, and chemoresistance (Wong and Yu, 2019). Although precise regulation of intestinal microbes for CRC treatment is not available clinically, microbiome intervention therapy, such as fecal microbiota transplantation and target-specific drugs, has caused wide attention. Specially, compared with chemotherapy drugs, natural products are potential lead compounds for new drug development with low toxicity and high efficiency.

Hundreds of microbes are related to CRC, yet only several of them have been studied in detail, including *Fusobacterium nucleatum* (*F. nucleatum*), *Bacteroides fragilis*, and certain strains of *Escherichia coli* (Zhou et al., 2020). *F. nucleatum* is an oral anaerobic bacteria and can cause opportunistic infections; however, Castellarin et al. reported that it was prevalent in human colorectal carcinoma for the first time (Castellarin et al., 2012). As a mutualist, infectious agent, and oncogenic microorganism, *F. nucleatum* was reported to be more enriched in CRC tissues of patients with recurrence and strongly associated with shorter recurrence-free survival (RFS) (Yu et al., 2017). It can promote colorectal carcinogenesis, exhibiting increased virulence in CRC when compared with the normal tissues (Rubinstein et al., 2013). Furthermore, *Fusobacterium* is associated with liver metastases from primary human colorectal cancers and positive lymph node metastasis (Bullman et al., 2017; Chen et al., 2020). Also, *F. nucleatum* helps tumors to construct an immune microenvironment, such as expansion of myeloid-derived immune cells and low tumor stromal CD3 lymphocytes, and is significantly associated with MSI-high (mismatch repair deficiency) independent of CIMP (CpG island methylator phenotype) and BRAF mutation status (Kostic et al., 2013; Mima et al., 2016; Borowsky et al., 2021). Recently, evidence revealed that high amounts of *F. nucleatum* were responsible for drug-resistance in CRC, including 5-fluorouracil and cisplatin (Zhang et al., 2019). Over the last decades, the carcinogenic mechanism of this pathogen is gradually elucidated. *F. nucleatum* adheres to and invades CRC cells via its unique FadA adhesin, binding to E-cadherin and forming FadA–E-cadherin–Annexin A1-β-catenin complex in cancerous cells. It can also induce oncogenic and inflammatory responses (Rubinstein et al., 2013). Fusobacterial Fap2 can recognize Gal–Gal–NAc to localize to CRC through a hematogenous route and increase proliferation of cancer cells (Yang et al., 2017). Moreover, *F. nucleatum* can promote distant metastases and chemoresistance *via* TLR4 and MYD88 innate immune signaling and autophagy pathway, providing target for clinical management as well as new drug development (Bullman et al., 2017; Yu et al., 2017). Recently, Yeoh and his colleagues found that southern Chinese populations harbor non-nucleatum Fusobacteria, being absent in western and rural populations (Yeoh et al., 2020). However, changes in gut microbiome, especially *F. nucleatum* in CRC patients in Northwest China, are rarely studied, which is an area with higher incidence of gastrointestinal tumors.

Apart from microbiota disorders, targeting and regulating pathogenic bacteria remain another issue. Treatment by traditional broad-spectrum antibiotics may point to both pathogen and other microbiota. Chemotherapy drugs will cause serious side effects and lead to chemotherapy resistance induced by some bacteria. For example, although metronidazole can decrease *Fusobacterium* load, it targets a range of anaerobic bacteria (Bullman et al., 2017). Recently, researchers reported that metformin relieved the symptoms induced by *F. nucleatum* administration in *APC* ^Min/+^ mice, rescuing *F. nucleatum*-induced tumorigenicity. It also corroborates that *Fusobacterium*-abundant CRC will benefit from non-antibiotic antifusobacterial therapy (Yu et al., 2017; Huang et al., 2020). Furthermore, it was reported that tungstate treatment selectively inhibited molybdenum-cofactor-dependent microbial respiratory pathways to ameliorate colitis (Zhu et al., 2018). Therefore, regulation of intestinal microbes is promising and will facilitate personalized cancer treatment.

Vanillin (4-hydroxy-3-methoxybenzaldehyde) is derived from the orchid *Vanilla planifolia* and used as one kind of flavor in various industries, such as ice creams and pharmaceuticals. Vanillin and its derivatives have different bioactive properties and are changing from a popular flavor to therapeutic molecules (Arya et al., 2021). It was found that vanillin could inhibit respiration of *E. coli* and *L. innocua*, increase the permeability of cell membranes, and cause more severe membrane damage after hydroxyl modification (Fitzgerald et al., 2004). Additionally, when combined with conventional antibiotics, vanillin could modulate the action of antibiotics against resistant bacteria, including *E. coli*, *Staphylococcus aureus*, and *Pseudomonas aeruginosa* (Bezerra et al., 2017). Furthermore, our previous studies showed that vanillin derivatives 4-[1H-imidazo(4,5-f) (1,10)phenanthrolin-2-yl]-2-methoxyphenol and 2-[1H-imidazo(4,5-f) (1,10)phenanthrolin-2-yl]-6-methoxyphenol (IPM711 and IPM712) could inhibit the growth, invasion, and migration of HT29 and HCT116 cells through the Wnt/β-catenin signaling pathway. After hydroxyl modification, derivative IPM712 showed better anticancer activity than 5-Fu and low toxicity at therapeutic concentrations. Further mechanism research demonstrated that it could affect the function of the PI3K/AKT signal pathway (Ma et al., 2020). However, whether vanillin derivatives can rescue opportunistic pathogen-induced (especially *F. nucleatum*) colorectal tumorigenesis deserves further study.

With this background, fecal samples of CRC patients and healthy people were used to explore the changes in intestinal microbial community and diversity. In addition, we tested the antibacterial activity of vanillin derivatives against *F. nucleatum* as well as its potential mechanism. Results indicated that IPM711 and IPM712 are potential novel agents for colorectal cancer treatment.

## Materials and Methods

### Sample Collection

Fecal samples were collected from the First Hospital of Lanzhou University and the Second Hospital of Lanzhou University. Those patients above 18 years old who went through colonoscopy examination were selected by reading medical records, including CRC, polypus, and healthy cases. The exclusion criteria were as follows: 1) had antibiotics for treatment in the last 3 months; 2) on a vegetarian diet; 3) experiencing surgeries in the last 3 months; 4) inflammatory bowel disease (IBD), Crohn’s disease, and diarrhea; 5) had any other cancer history; and 6) had received radiotherapy or chemotherapy. Samples were collected either prior to colonoscopy or 1–2 weeks after colonoscopy, placed on ice, transported to the laboratory, and stored at −80°C immediately. The study protocol was approved by the Ethical Review Board of the hospital (LDYYLL 2020-241 and 2021A-152). Informed consent was obtained from all study patients.

### 16S rRNA Sequencing and Bioinformatics Analysis

Genomic DNA was extracted using the TIANamp Stool DNA Kit (TIANGEN BIOTECH) following the manufacturer’s instructions. Quality and quantity of DNA were verified with NanoDrop 2000 and 1% agarose gel. Bacterial V3–V4 regions of the 16S rRNA gene were amplified with universal primers 343F and 798R. PCR products were purified with AMPure XP beads (Agencourt) and quantified using Qubit dsDNA assay kit.

Paired-end reads were then preprocessed using Trimmomatic software and assembled using FLASH software (Reyon et al., 2012; Bolger et al., 2014). Sequences were performed further denoising as follows: reads with ambiguous, homologous sequences or below 200 bp were abandoned. Reads with 75% of bases above Q20 were retained. Then reads with chimera were detected and removed using the QIIME software (version 1.8.0) (Caporaso et al., 2010). Clean reads were subjected to clustering to generate operational taxonomic units (OTUs) using the Vsearch software with 97% similarity cutoff (Edgar et al., 2011). Species richness was represented by observed species, while community diversity was expressed using phylogenetic diversity (Faith, 1992).

### Bacteria and Cell Culture

*F. nucleatum* was purchased from the China General Microbiological Culture Collection Center (CGMCC) and cultured with EG medium (Euglena gracilis medium) in an anaerobic box at 37°C, and the number of *F. nucleatum* was determined by the standard curve. HCT116 cell line was cultured in RPMI-1640 medium supplemented with 10% FBS and 1% antibiotics at 37°C and 5% CO_2_. *F. nucleatum* and cancer cells (multiplicity of infection, MOI = 100) were cocultured for 2 h at 37°C and treated with compounds at the half maximal inhibitory concentration (IC_50_) for 48 h. Experiments were performed with cells of passage numbers 4 to 20. Experiments were repeated three times.

### Minimum Inhibitory Concentrations and Minimum Bactericidal Concentrations Test

*F. nucleatum* was harvested at logarithmic growth period (24–48 h), and the concentration of the bacterial solution was adjusted to 10^6^ CFU/ml. IPM711 and IPM712 were diluted to 40, 80, 120, 160, and 200 μM with EG medium. Then 50 μl of compound solution and 50 μl of bacterial solution were added to 96-well plates and mixed for 10 min. After 48 h of anaerobic culture at 37°C, concentrations of clarified wells were determined as the minimum inhibitory concentrations. Finally, 50 μl of culture solution was collected from each clarified well and spotted onto the EG plates, and concentrations of plate without colonies were determined as the minimum bactericidal concentrations (MBC). Experiments were repeated three times.

### MTT Assay for Inhibition

MTT was used to determine the inhibition, and L-cysteine was removed from the medium formula. *F. nucleatum* was harvested and diluted to an optical density at 600 nm (OD_600nm_) of 0.2. Next, 50 μl of bacterial solution and the same volume of compound solution were added to the 96-well plate with the final concentration at 20, 40, 60, 80, and 100 μM, which were cultivated for 24 h under anaerobic conditions at 37°C. Finally, absorbance (Abs) values were detected at 490 nm, and the cell viability was calculated according to the formula = 100% × (sample Abs)/(control Abs). Penicillin–streptomycin and EG medium were set as positive and negative controls, respectively.

### Effects on Bacterial Membrane Permeability

Propidium iodide (PI) was used to study the effect on bacterial membrane permeability, which could only bind to the nucleus through broken cell membranes. First, the density of *F. nucleatum* was diluted to 0.4 at OD_600nm_. Then 1 ml of bacterial solution and compound solution were mixed and incubated for 24 h under anaerobic conditions at 37°C. Next, the solution was centrifuged for 2 min at 10,000 rpm and washed twice with 1× phosphate-buffered solution (PBS). Finally, *F. nucleatum* was stained with 50 mg/ml of PI solution for 20 min. To determine the total bacteria, it was fixed using 4% paraformaldehyde. Measurements were made using fluorescence microscope (BX53, Olympus). Experiments were repeated three times.

### Effects on Bacterial Integrity

The density of *F. nucleatum* was diluted to 0.4 at OD_600nm_ and incubated with compounds (MIC). Three hours later, the bacterial suspension was centrifuged for 5 min at 10,000 rpm and fixed with 2.5% glutaraldehyde for 4 h at 4°C. Then it was centrifuged to remove the supernatant and dehydrated with 20%, 50%, 80%, and 100% (twice) ethanol for 15 min each time. Next, the bacterial solution was adjusted to a suitable concentration and frozen dried for 4 h. Finally, a layer of gold was sprayed onto the surface, and *F. nucleatum* was observed using a scanning electron microscope (SEM, JSM-6701F, JEOL).

### Real-Time Quantitative PCR

After coculture with *F. nucleatum*, HCT 116 cells (1 × 10^6^ cells) were treated with IPM711 and IPM712 at the concentration of IC_50_ (6.69 and 1.40 μM, respectively) for 48 h. Total RNA was extracted using RNAsimple Total RNA Kit and subjected to reverse transcription with FastKing gDNA Dispelling RT SuperMix (TIANGEN BIOTECH) following the manufacturer’s instructions. Quantitative PCR (qPCR) assay was used to measure the relative abundance of the gene. Each 10 μl of reaction volume contained 1× SuperReal PreMix Plus (SYBR Green, TIANGEN BIOTECH), 0.3 μM of each primer, 1× ROX reference dye. Primers used here are listed in Table 1 and the conditions were as follows: 15 min at 95°C, 40 cycles of 15 s at 95°C, and 32 s at 60°C. A melting curve step was run after the qPCR reaction to verify the specificity of each primer. All qPCR amplifications were run on the Applied Biosystems QuantStudio 5 Real-Time PCR Systems (Applied Biosystems, USA). The relative abundance was calculated using 2^−ΔΔCt^ method. All experiments were performed in triplicate.

**TABLE 1:** Primers used for qPCR in this study.

Name	Sequences
GAPDH-F	5′-GGA​GCG​AGA​TCC​CTC​CAA​AAT-3′
GAPDH-R	5′-GGC​TGT​TGT​CAT​ACT​TCT​CTC​ATG​G-3′
RASA-F	5′-GTG​GCC​GGT​GCT​GCT​GTT​GC-3′
RASA-R	5′-TGG​CCA​CCT​GTT​CCT​CCT​CGT​ATT-3′
MYD88-F	5′-GCC​GCC​GGA​TGG​TGG​TGG​TTG​T-3′
MYD88-R	5′-TTG​GTG​CAG​GGG​TTG​GTG​TAG​TCG-3′
E-cadherin-F	5′-ATT​TTT​CCC​TCG​ACA​CCC​GAT-3′
E-cadherin-R	5′-TCC​CAG​GCG​TAG​ACC​AAG​A-3′
PI3KCA-F	5′-TGA​AGC​ACC​TGA​ATA​GGC​AAG​TCG-3′
PI3KCA-R	5′-TCT​GGT​CGC​CTC​ATT​TGC​TCA​AC-3′
β-catenin-F	5′-CAA​CTA​AAC​AGG​AAG​GGA​TGG​A-3′
β-catenin-R	5′-CTA​TAC​CAC​CCA​CTT​GGC​AGA​C-3′
AKT-F	5′-AGC​GAC​GTG​GCT​ATT​GTG​AAG-3′
AKT-R	5′-GCC​ATC​ATT​CTT​GAG​GAG​GAA​GT-3′
GSK-3β-F	5′-GAC​TAA​GGT​CTT​CCG​ACC​CC-3′
GSK-3β-R	5′-AAG​AGT​GCA​GGT​GTG​TCT​CG-3′

### Western Blot

For Western blot experiments, 1 × 10^6^ cells were seeded into six-well plates. The coculture and treatment were the same as described above. Then the total proteins were extracted with RIPA buffer containing 1% PMSF and detected with Brasford assay. Proteins of different sizes were separated using 10% sodium dodecyl sulfate-polyacrylamide gel electrophoresis (SDS-PAGE). Afterward, proteins were transferred onto a 0.22-μm PVDF membrane and incubated with Tris buffer containing 5% nonfat milk, which was further incubated with primary antibodies, including E-cadherin (#bs-1519R, 1:1,000, Bioss) and β-catenin (#bs-1165R, 1:1,000, Bioss), and secondary antibody (#bs-40295G-HRP, 1:5,000, Bioss) for 12 and 1 h, respectively. Subsequently, the expression of proteins was analyzed using the chemiluminescence analysis system and ImageJ 1.43 software. GAPDH (#bs-10900R, 1:5,000, Bioss) was used as a control for whole-cell lysates. All experiments were performed in triplicate.

### Migration Assay

HCT116 cells (1 × 10^6^ cells) were seeded into six-well plates and cocultured with *F. nucleatum* for 2 h under anaerobic conditions. Then cells were washed with PBS three times and seeded at a density of 1 × 10^5^ cells/0.1 ml in RPMI-1640 (1% FBS) into the upper chambers (8-μm pore size, Corning). The lower chamber was filled with 500 μl of media with 20% FBS. After incubation for 48 h, cells were fixed with 4% paraformaldehyde for 20 min at room temperature, and then stained with 0.1% crystal violet for 20 min. Migrating cells were visualized by microscopy at ×40 magnification and counted with the ImageJ 1.43 software. All experiments were performed in triplicate.

### Statistical Analysis

Transcriptome data from colorectal cancer and the adjacent normal tissues infected with *F. nucleatum* in the public GEO datasets were used as external validation. Additionally, gene expression abundance, and overall survival of healthy people and CRC patients were analyzed using GEPIA (http://gepia.cancer-pku.cn/) (Tang et al., 2017).

All statistical analysis were performed with the OriginPro software (OriginLab Corporation, United States) and R Project for Statistical Computing environment. qPCR data were analyzed using QuantStudio™ Design and Analysis Software (Thermo Fisher Technology). Statistical differences between groups were analyzed using t-test. All results were expressed as the mean ± standard deviation of independent experiments. A value of *p* < 0.05 was considered statistically significant.

## Results

### Changes of Microbial Community and Diversity

The overall study population consists of 22 CRC patients, 22 cases with different degrees of polypus, and 18 controls who went through colonoscopy but with no disease symptoms. The mean age of the cohort is 59.5 years old, and female subjects account for 40.0%. The number of valid tags of each sample was distributed between 26,376 and 37,118, with the average length distributed between 414.35 and 420.06 bp.

**FIGURE 1:**
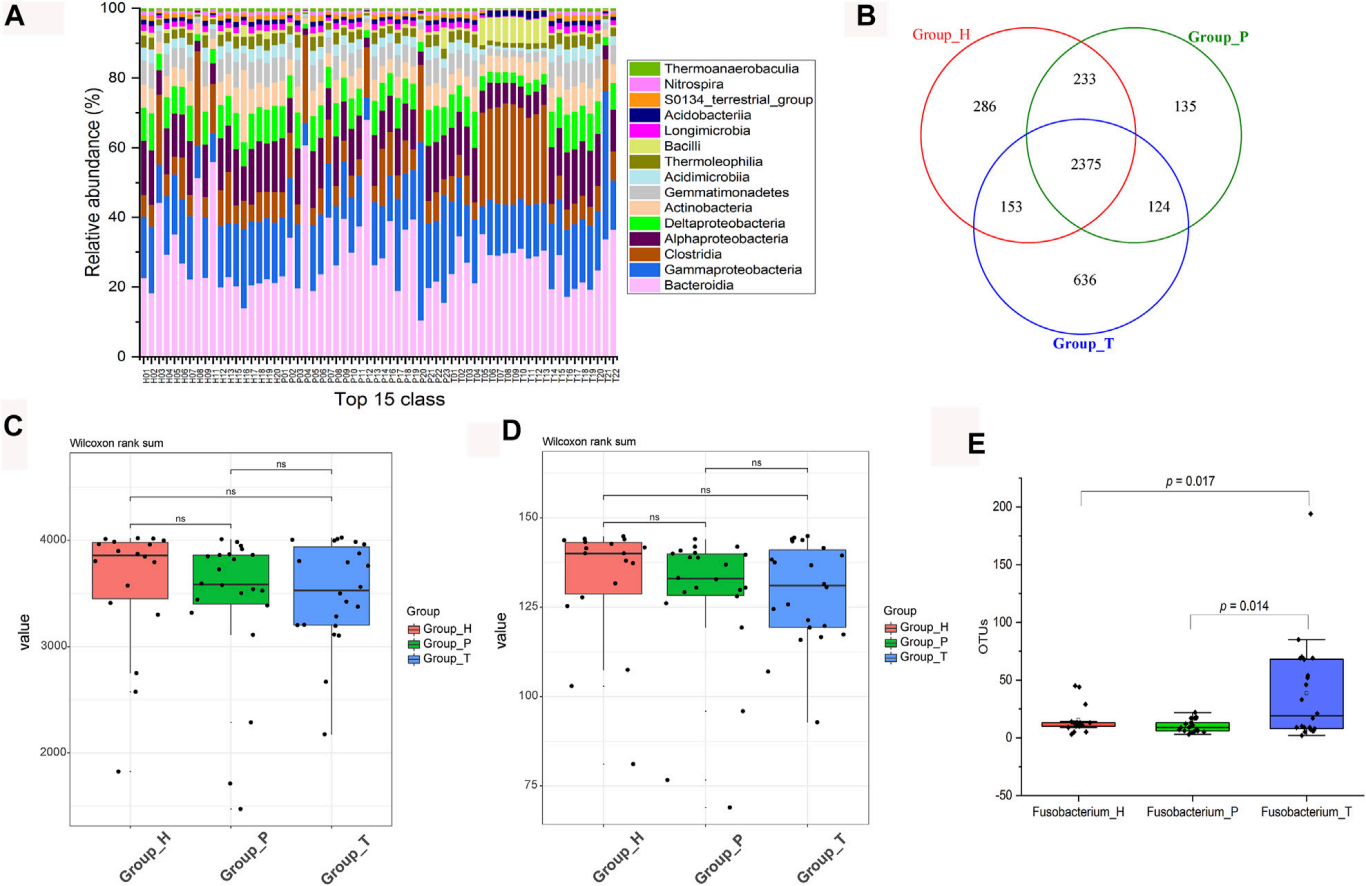
Changes in microbial community and diversity. Relative abundance of the top 15 classes (H, healthy people; P, polyp patients; T, CRC patients) **(A)**. Venn diagram of different groups **(B)**. Comparation among observed species **(C)**. Comparation among phylogenetic diversity **(D)**. Absolute abundance (OTU numbers) of *Fusobacterium* in different groups **(E)**.

To investigate the signature of the three groups, microbial community structure and alpha diversity were used for comparison. In CRC patients, the abundance of Clostridia and Bacilli was higher, while the healthy group contained more Gammaproteobacteria and Deltaproteobacteria (Figure 1A). Compared with the polyp group, the difference in composition of species between healthy and cancer groups was bigger (Figure 1B). In addition, both species richness and community diversity showed a stepwise decreased frequency from controls, to dysplasia and to cancers, indicating that the development of CRC was accompanied with depletion of gut microbes (Figures 1C, D). We further studied the change in *Fusobacterium* and found that its abundance gradually increased along the deterioration of the disease (the sample H06 was excluded according to the F-test) (Figure 1E).

### Antimicrobial Effect of IPM711 and IPM712

**FIGURE 2:**
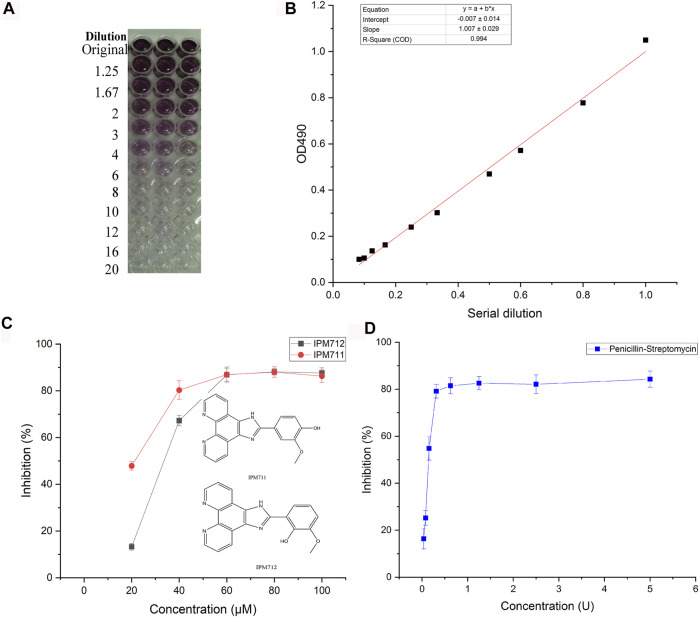
Anti-*F. nucleatum* activities of IPM711 and IPM712. Gradient changes in color in a 96-well plate at different *F. nucleatum* concentrations using the MTT method and corresponding standard curve at 490 nm **(A,B)**. Inhibition rates of IPM711 (red point), IPM712 (black point), and penicillin–streptomycin (blue point) against *F. nucleatum* **(C,D)**.

As one of the most prevailing pathogenic bacteria, *F. nucleatum* was used for antibacterial test of IPM711 and IPM712. Results showed that their MIC values were 80 and 60 μM, respectively. Both MBC values were 80 μM. Furthermore, MTT assay was applied for the detection of inhibition rate, and the standard curve showed that there was a good linear relationship between the number of viable bacteria and the absorbance (R^2^ > 0.99, Figures 2A, B). The inhibition rates of IPM711, IPM712, and antibiotics were 88.09%, 88.00%, and 87.25% at 100 μM/5 U (Figures 2C,D). Overall, IPM712 has a higher antibacterial activity, demonstrating that the change in the hydroxyl position reduces the steric hindrance and increases the activity, which is consistent with their antitumor effects.

### Morphology Changes of Bacterial Cells

**FIGURE 3:**
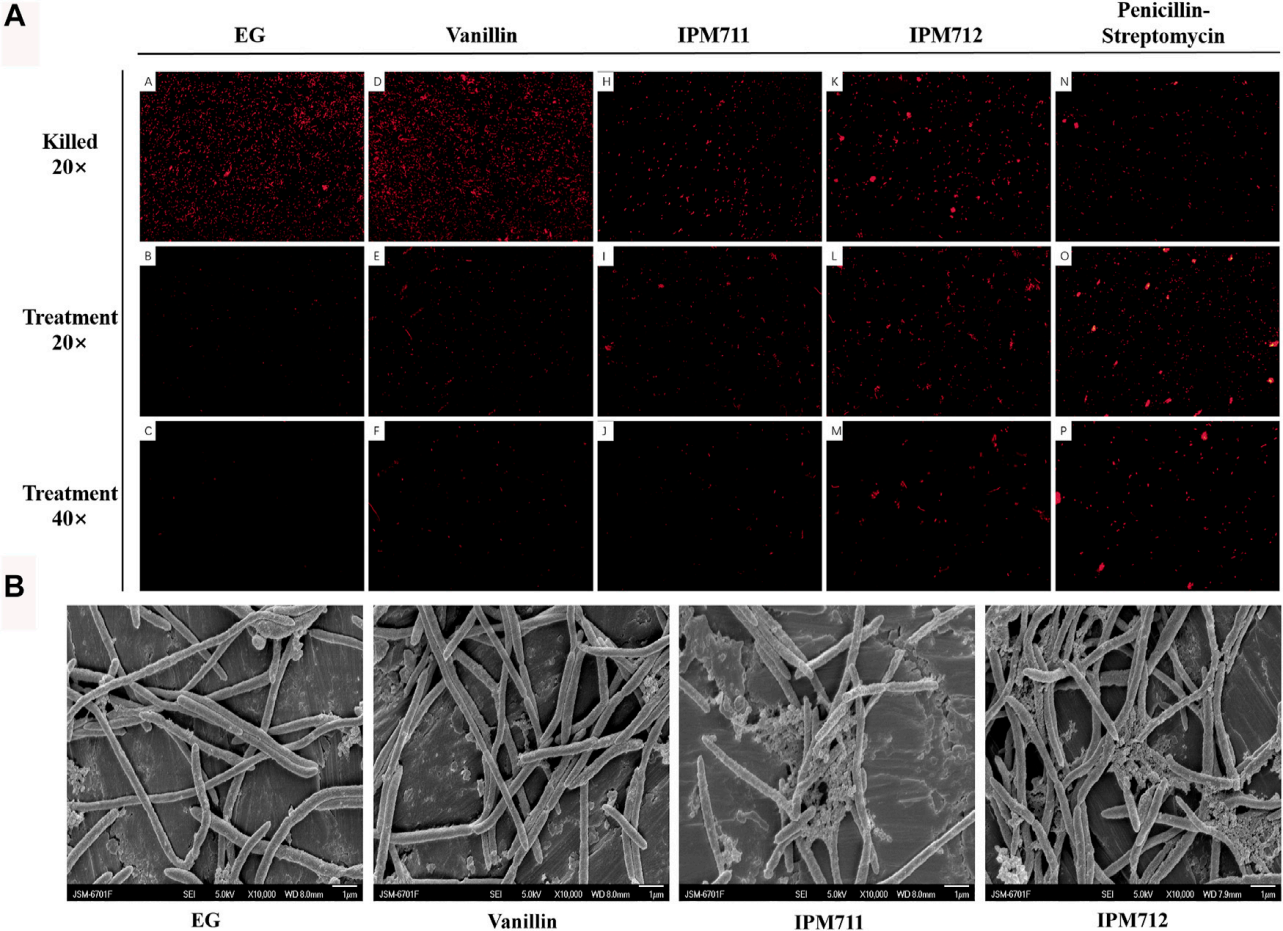
Fluorescence microscope observation after propidium iodide (PI) staining and bacterial morphology after exposure to vanillin and its derivatives. Observation (20×) after 4% paraformaldehyde fixation and stain (marked as “killed” on the first row). Observation (20×) after *Euglena gracilis* (EG) medium, vanillin, IPM711, IPM712, and penicillin–streptomycin treatments (the second row). Observation (40×) after treatment (the third row) **(A)**. Scanning electron microscope (SEM) observation after EG medium, vanillin, IPM711, and IPM 712 treatments **(B)**.

Based on the antibacterial activity results, PI staining and SEM were carried out to observe the bacterial morphology change after exposure to compounds. Paraformaldehyde-treated bacteria were used to display all the *F. nucleatum* in the different groups, and results showed a decreased number of *F. nucleatum* after treatment of compounds (Figure 3A). Additionally, compared with EG medium and vanillin groups, IPM711 and IPM712 groups had stronger fluorescence, indicating that they could strongly increase the permeability of cell membranes, which was similar to the penicillin–streptomycin group. SEM observation showed that the outer surface of *F. nucleatum* was complete and smooth after treatment with EG medium and vanillin, yet the bacteria became rough and some broke after treatment with IPM711 and IPM712 (Figure 3B). Results suggested that IPM711 and IPM712 could cause the breakage of bacterial cells and destroy the integrity, thus, exerting better antibacterial activity than vanillin.

### *F. nucleatum* Induce Colorectal Tumourigenesis

**FIGURE 4:**
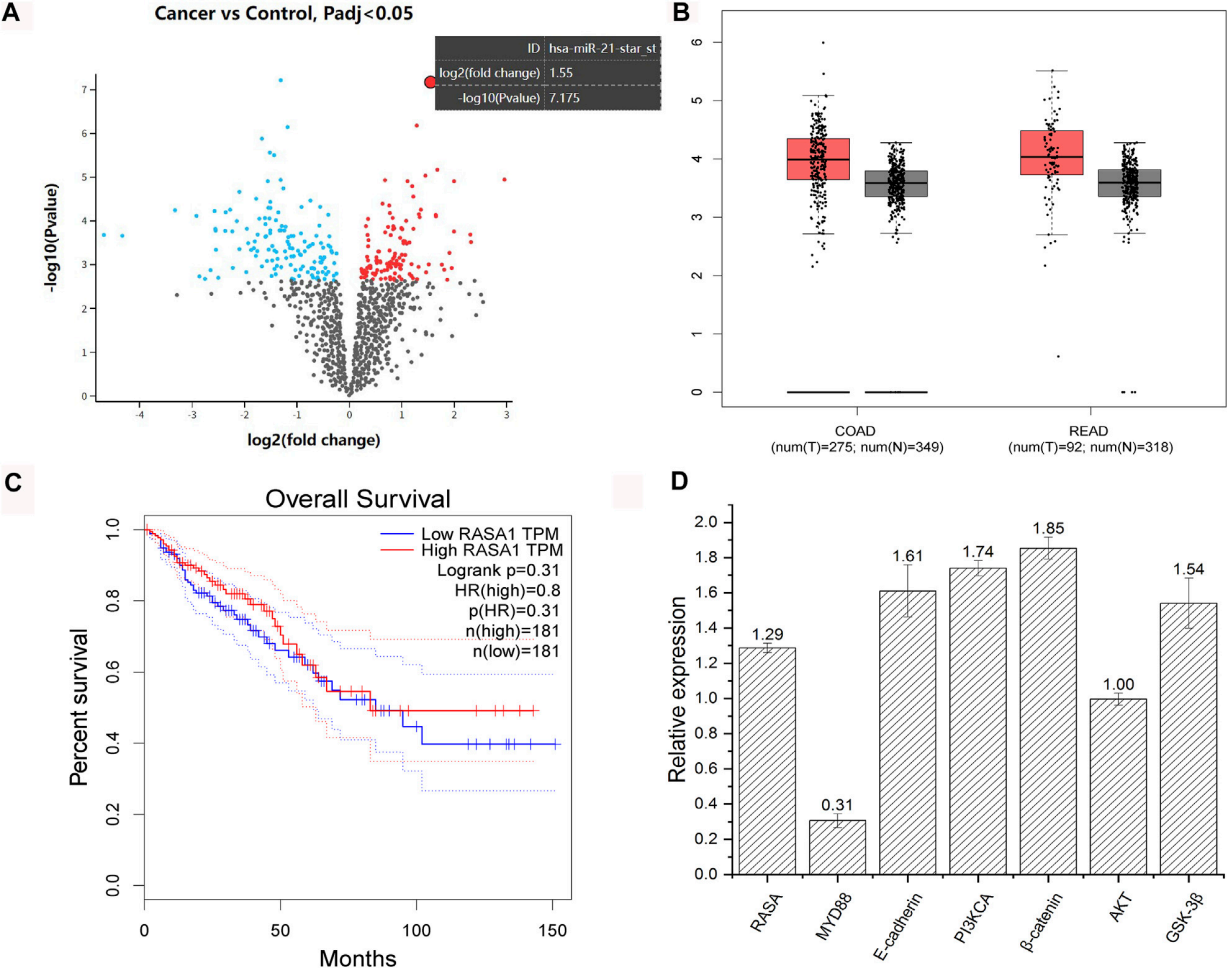
Analysis of GSE122182 dataset and validation with qPCR. Volcano map of gene expression of colorectal cancer and the adjacent normal tissues infected with *F. nucleatum* (red points represent upregulated genes and blue points represent downregulated genes) **(A)**. Expression difference of *RASA* gene between healthy people and CRC patients (COAD, colon cancer; RECD, rectal cancer; T, tumor patients; N, normal people) **(B)**. Survival curve of patients with high and low expression of *RASA* gene (obtained from GEPIA, http://gepia.cancer-pku.cn/) **(C)**. Changes in gene expressions of HCT116 after coculture with *F. nucleatum* (numbers represent the fold change in gene expression) **(D)**.

*F. nucleatum* was reported to be more abundant in CRC tissues, and expression data (GSE122182) of miRNA profile from colorectal cancer and the adjacent normal tissues infected with *F. nucleatum* showed that miRNA 21 was the most upregulated, whose corresponding gene is *RASA* (Figure 4A). Further analysis showed that the expression level of *RASA* was higher in CRC patients. Those patients with high *RASA* expressions displayed shorter survival period (GEPIA, http://gepia.cancer-pku.cn/, Figures 4B, C). In addition, after coculture of *F. nucleatum* and HCT116 cells, qPCR experiments showed that *F. nucleatum* induced upregulated expression of *RASA*, *PI3KCA*, *β-catenin*, *E-cadherin*, and *GSK-3β*, yet without any influence on the *AKT* gene. Meanwhile, expression of *MYD88* was downregulated (Figure 4D). Those genes were previously reported to be associated with apoptosis, invasion, and chemotherapy resistance of colorectal cancer.

### IPM711 and IPM712 Reverse *Fusobacterium nucleatum*-Induced Colorectal Tumorigenesis

**FIGURE 5:**
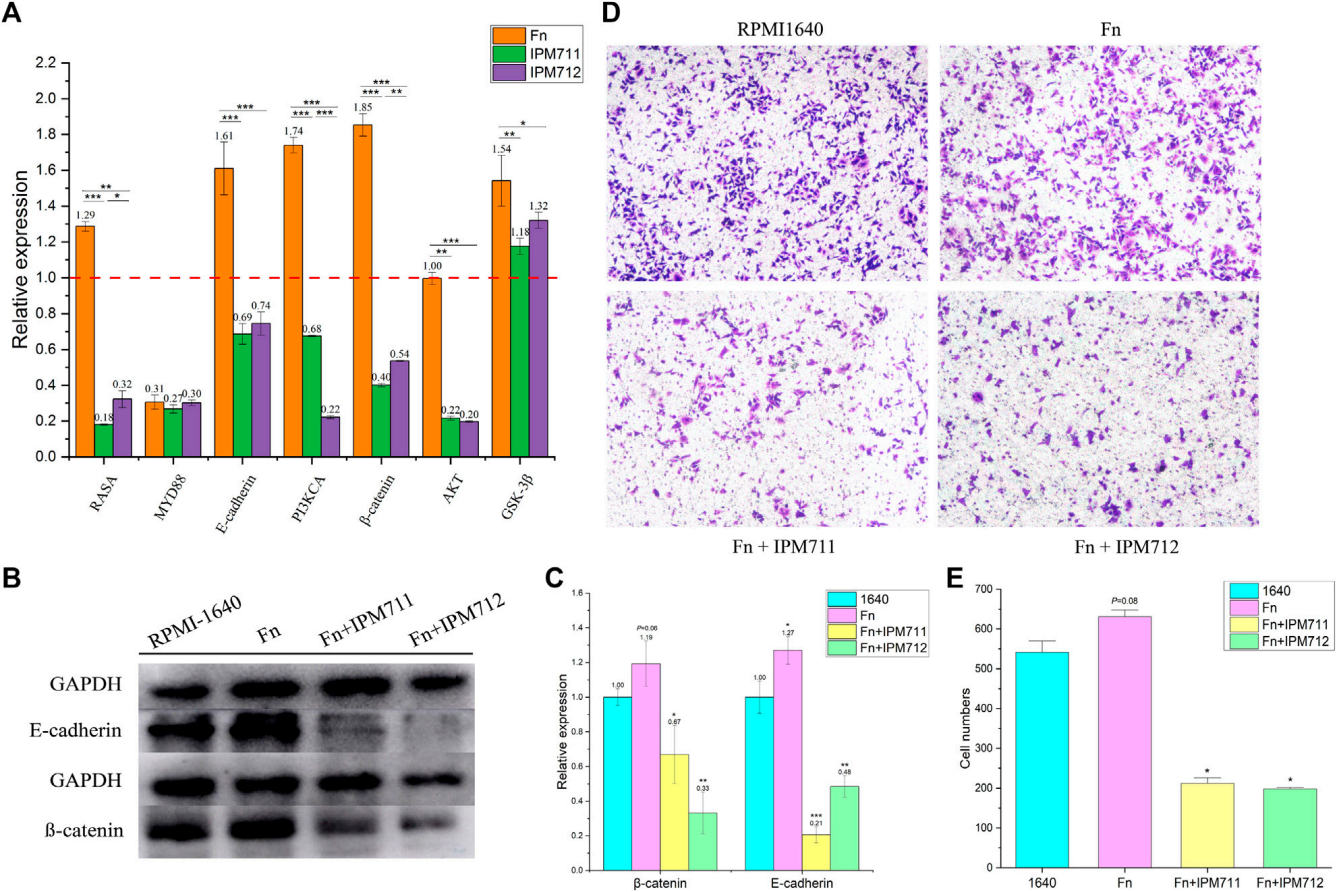
Changes in gene and protein expression. Changes in related genes of HCT116 after cocultivation with *F. nucleatum* (Fn, MOI = 100, 2 h) and treatments with IPM711 and IPM712 at the concentration of IC_50_ **(A)**. Changes in E-cadherin and β-catenin proteins of HCT116 after cocultivation with *F. nucleatum* (MOI = 100, 2 h) and treatments with IPM711 and IPM712 (IC_50_) **(B)**. Statistical analysis, *p < 0.05, **p < 0.01, ***p < 0.001 compared with control group **(C)**. Characterization of migratory capacity of HCT116 cells after treatment with RPMI-1640, *F. nucleatum*, *F. nucleatum* and IPM711, and *F. nucleatum* and IPM712 **(D)**. Statistical analysis, *p < 0.05 compared with control group **(E)**.

To verify whether IPM711 and IPM712 are able to treat CRC after *F. nucleatum* infection, compound treatments were performed after coculture of *F. nucleatum* and HCT116 cells. qPCR results showed that IPM711 and IPM712 could downregulate the overexpressed *RASA* gene significantly, but without any effect on *MYD88* gene, suggesting that IPM711 and IPM712 might rescue *F. nucleatum*-induced chemoresistance somehow instead of the TRL4/MYD88 pathway. Moreover, the expression of *E-cadherin*, *PI3KCA*, *β-catenin*, *AKT*, and *GSK*-*3β* were downregulated, indicating that IPM711 and IPM712 could reduce the proliferation and migration of cancer cells as well as the adhesion of *F. nucleatum* (Figure 5A). Western blot results also showed that E-cadherin and β-catenin proteins were downregulated after IPM711 and IPM712 treatments, corroborating the gene expression changes (Figures 5B, C). Finally, migration assay proved that IPM711 and IPM712 still exerted anticancer effect in the case of *F. nucleatum* infection (Figure 5D).

## Discussion

**FIGURE 6:**
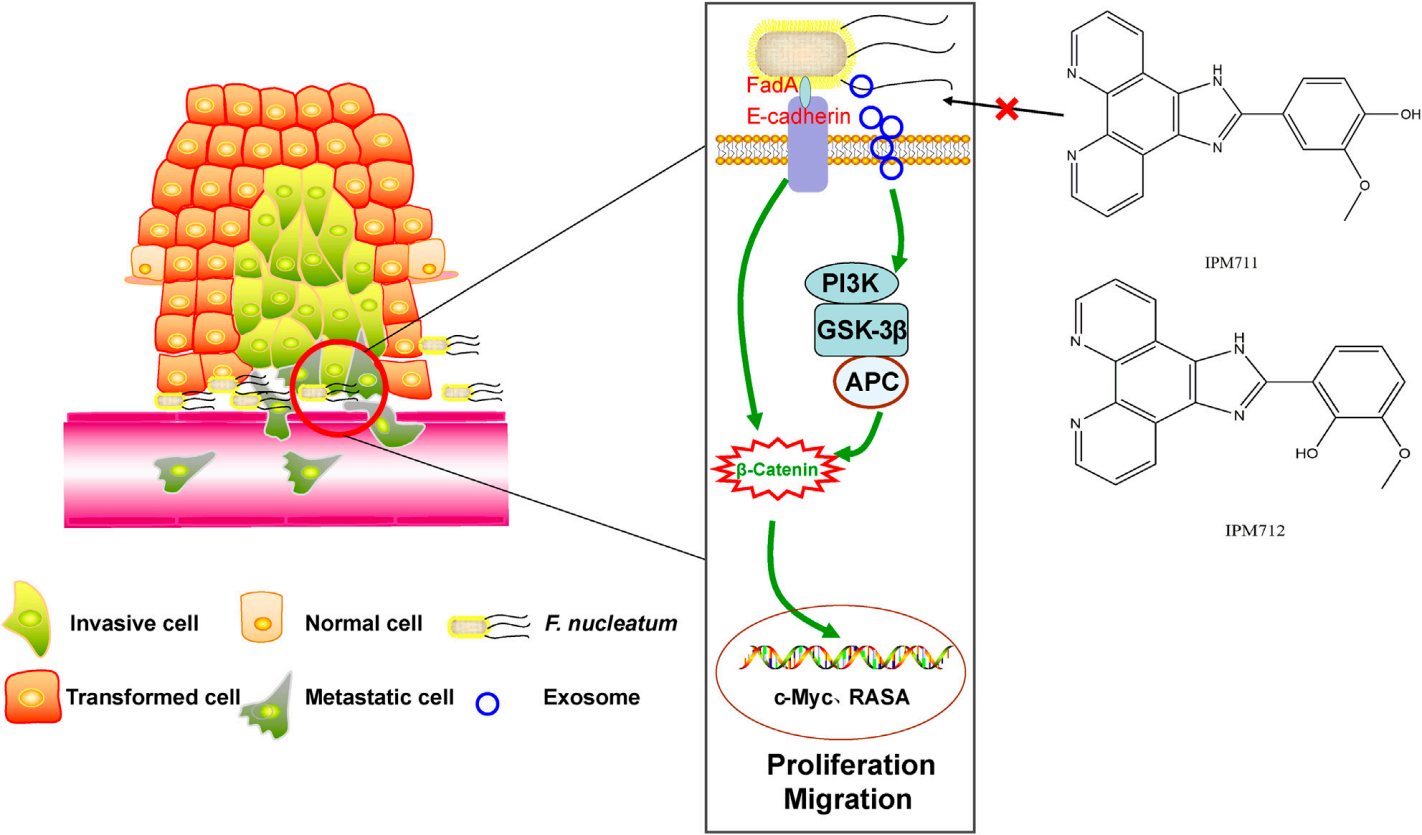
Vanillin derivatives reverse *F. nucleatum*-induced proliferation and migration of CRC through the E-cadherin/β-catenin pathway.

Gut microbiome dysbiosis and increase in *Fusobacterium* are common in CRC patients and verified in our sample set in Northwest China for the first time. In addition, vanillin derivatives, IPM711 and IPM712, showed better anti-*F. nucleatum* activity after structural modification. Furthermore, intervention experiments *in vitro* indicated that they were effective for *F. nucleatum*-infected CRC cells by inhibiting proliferation and migration through the E-cadherin/β-catenin pathway (Figure 6).

Disorders of the gut microbiome have been acknowledged as one important factor of CRC development, and panels of different microbes can be used as biomarkers for early diagnosis. People from different countries or areas carry different microbiome characteristics, which may result from their unique regional environmental factors. Fusobacteria is not relatively abundant in healthy people but common in CRC patients. However, *Fusobacterium* are prevalent in most CRC populations in China, though several *Fusobacterium* taxa are comparatively more abundant in southern Chinese populations (Yang et al., 2020; Yeoh et al., 2020). Northwest China, especially Gansu and Ningxia, are areas with a high incidence of gastrointestinal tumors (Zhang et al., 2021), yet the gut microbiome changes in CRC patients in these areas are unclear. Our results showed that patients’ intestinal microbiome gradually changed with the development of the disease, manifested as an increase in harmful bacteria, a decrease in beneficial bacteria, and a gradual decline in overall diversity. Moreover, predictions in the Kyoto Encyclopedia of Genes and Genomes (KEGG) displayed that pathways of CRC patients were mainly related with the calcium signaling pathway, various types of N-glycan biosynthesis, the GnRH signaling pathway, endocytosis, and Fc gamma R-mediated phagocytosis, while the main functions in healthy people included glycolysis/gluconeogenesis, cysteine and methionine metabolism, primary immunodeficiency, and peptidoglycan biosynthesis (Supplementary Figure S1), suggesting that the dysregulation in the gut microbiome might be accompanied by changes in metabolic function. Most importantly, *Fusobacterium* is more abundant in CRC patients in Northwest China, whereas the specific taxa and associated functions need further exploration using powerful tools, such as metagenomic and metatranscriptome sequencing. Another limitation is that changes in the microbial community or *Fusobacterium* are based on the microorganisms shed into the feces. Although fecal samples are more convenient for collection and noninvasive for early diagnosis (Wong et al., 2017), they may contain the bacteria throughout the digestive tract. Therefore, tissue samples are needed to further verify those changes.

*F. nucleatum* can produce volatile sulfur compounds, such as H_2_S and CH_3_SH, being considered to be related to oral halitosis initially. Subsequently it was found to be prevalent in human colorectal carcinoma. To reduce these kinds of bacteria, many natural products and active molecules had been tested to inhibit *F. nucleatum*, including Labrador tea, peppermint, winter savory essential oils, tea polyphenols, wild blueberry polyphenols, *Litsea japonica* leaf extract, curcumin, dietary (poly) phenols, graphene oxide, and macrolides (Nagaoka et al., 2013; Ben Lagha et al., 2015; He et al., 2015; Shahzad et al., 2015; Izui et al., 2016; Ben Lagha et al., 2017; Yun et al., 2018; Ben Lagha et al., 2020), indicating that phenolic compounds possess superior activity against *F. nucleatum*. A study evaluated the resistance of natural and synthetic phenolic compounds to oral bacteria, finding that lipophilicity and steric effect were two key factors. They acted as non-ionic surfactants and destroyed the lipoprotein interface (Greenberg et al., 2008). Similarly, after structural modification, ortho-hydroxyl groups enhanced the antibacterial activity of vanillin derivatives, which was consistent with the anticancer effect (Ma et al., 2020). Currently, narrow-spectrum antibiotics targeting *F. nucleatum* are not available, thus, combining conventional chemotherapy (5-fluorouracil, cisplatin, etc.) with well-tolerated natural compounds may help to better respond to treatment and improve patients’ quality of life. Recently, a systematic review summarized natural compounds possessing anti-CRC activities and proposed advantages of combination therapy, including reduction of resistance to cancer drugs, alleviation of the toxic burden on the patient’s body, and increased sensitivity to chemotherapeutic drug by targeting multiple metabolic pathways (Rejhová et al., 2018). Therefore, natural products, such as vanillin derivatives, are potential candidates for the treatment of *F. nucleatum*-infected CRC, which will lead to chemotherapy resistance to 5-fluorouracil and cisplatin (Yu et al., 2017; Zhang et al., 2019).

Results of qPCR and Western blot suggested that IPM711 and IPM712 inhibited the expression of E-cadherin (the occurrence of double bonds might result from the slightly poor specificity of polyclonal antibody) and β-catenin, which were indispensable for the adherence of *F. nucleatum* to HCT116 cells. E-cadherin mediates bacterial adhesion to mammalian cells, and β-catenin (also called CTNNB) is necessary for the creation and maintenance of epithelial cell layers by regulating cell growth and adhesion between cells, both of which are overexpressed in tumor tissues (Supplementary Figure S2). Previous studies demonstrated that *F. nucleatum* could stimulate growth of CRC cells through its FadA adhesion, which binds to E-cadherin and activated β-catenin signaling (Rubinstein et al., 2013). Another research found that FadA, E-cadherin, Annexin A1, and β-catenin could form a complex in cancerous cells, and FadA adhesin from *F. nucleatum* upregulated Annexin A1 expression through E-cadherin (Rubinstein et al., 2019). The protein encoded by the *RASA* gene is part of the GAP1 family of GTPase-activating proteins, thereby, allowing control of cellular proliferation and differentiation. Upregulation of miR-223 was associated with the downregulation of *RASA* in CRC tissues, and C/EBP-β-activated miR-223 improved colorectal cancer cell growth and stimulated tumorigenesis by targeting *RASA* in CRC (Sun et al., 2013; Sun et al., 2015). Furthermore, overexpression of *RASA* could abolish the promotive effects of exosome-transmitted miR-335-5p on CRC cell migration, invasion, and epithelial–mesenchymal transition (Sun et al., 2021). Recently, it was reported that *F. nucleatum* could activate TLR4 signaling to MYD88 and reduce levels of *RASA*, thus, leading to chemoresistance (Yang et al., 2017). IPM711 and IPM712 could significantly downregulate the expression *RASA* gene and might relieve chemoresistance. Furthermore, IPM711 and IPM712 also changed the upregulation of *GSK-3β* induced by *F. nucleatum*, which was recently proved to be associated with exosomes derived from *F. nucleatum* (Guo et al., 2021), suggesting that they might inhibit the proliferation and migration of CRC cells by influencing the exosomes. With the discovery of the carcinogenic mechanisms of *F. nucleatum*, treatment of CRC by targeting the bacteria has become a feasible strategy. Natural products are important source of drug development with low toxicity and high efficiency. It is of great significance to develop natural medicines that can resist both colorectal cancer and pathogenic bacteria.

## Data Availability

The datasets presented in this study can be found in online repositories. The names of the repository/repositories and accession number(s) can be found below. The datasets (16S rRNA sequencing) for this study can be found in the NCBI Short Read Archive (PRJNA763872).
