# Supplementary material for: Vanillin Derivatives Reverse Fusobacterium nucleatum-Induced Proliferation and Migration of Colorectal Cancer Through E-Cadherin/β-Catenin Pathway
Source: Front Pharmacol. 2022 Mar 4;13:841918. doi: 10.3389/fphar.2022.841918 (PMC8931468; doi:10.3389/fphar.2022.841918)
Supplement: Supplementary file 1 [file DataSheet1.docx]

Supplementary Material

# Supplementary Figures


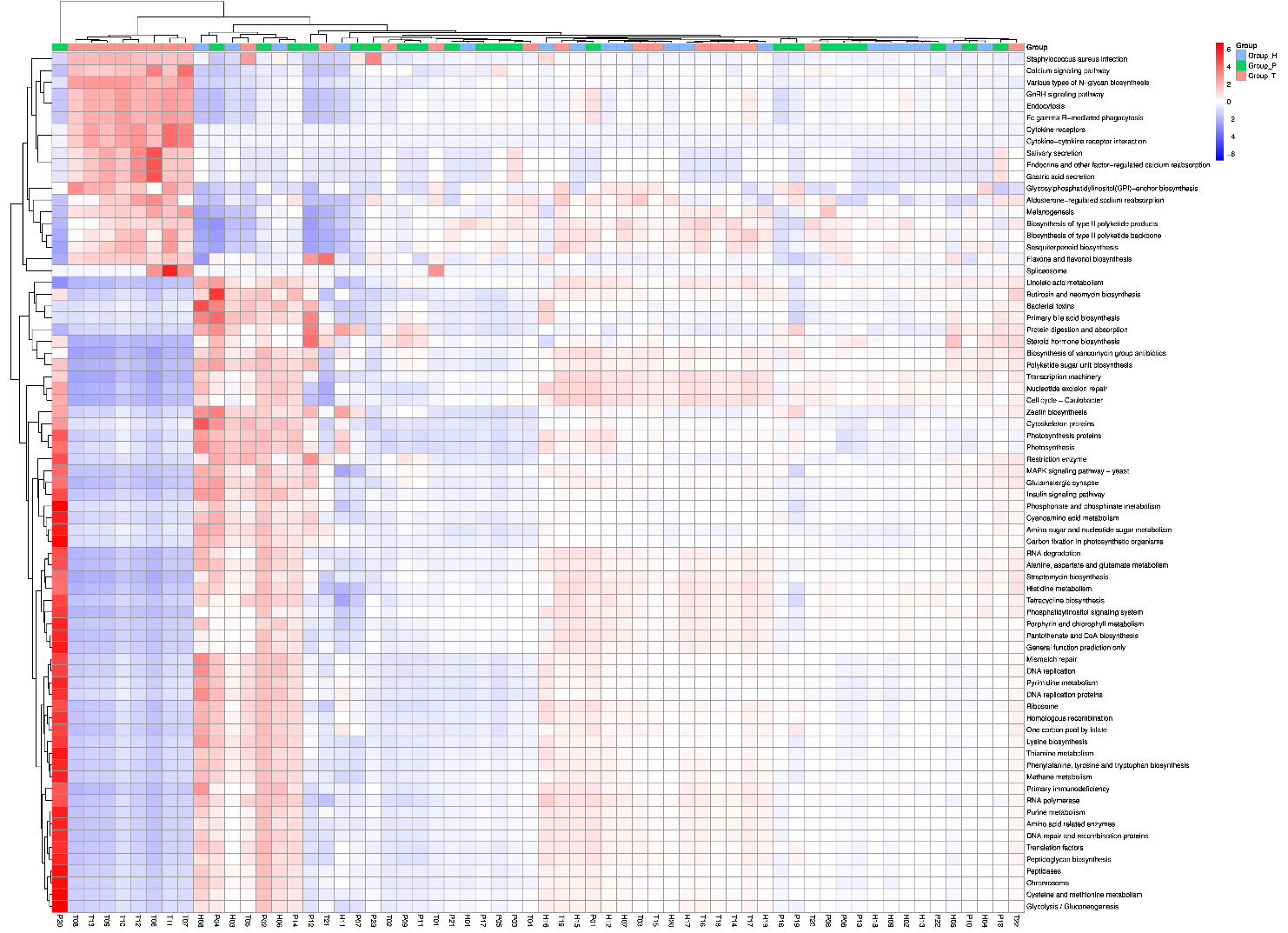


**Supplementary Figure 1.** Kyoto Encyclopedia of Genes and Genomes (KEGG) function prediction based on 16S rRNA sequencing.


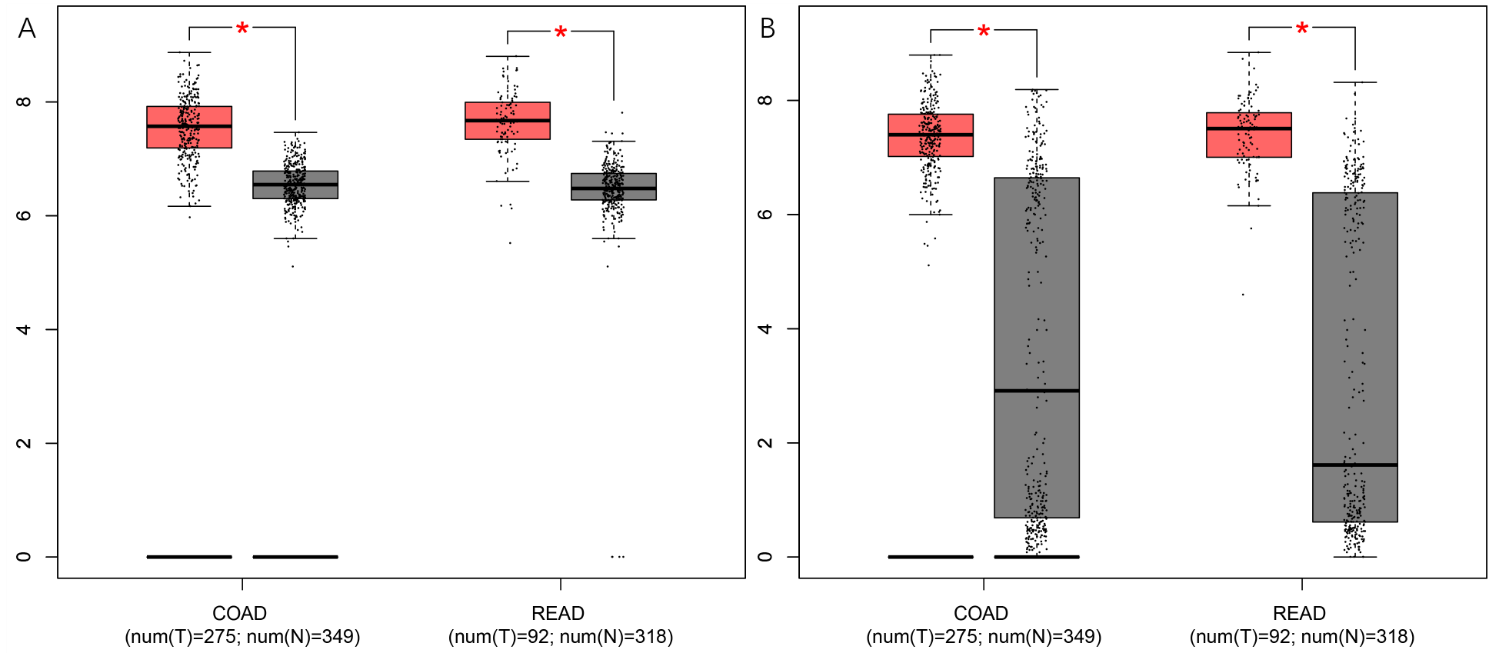


**Supplementary Figure 2.** Relative expression of *β-catenin* (A) and *E-cadherin* (B) in tissues of healthy people and CRC patients (COAD, colon cancer; RECD, rectal cancer; T, Tumor patients; N, normal people).
